# 570. Pharmacokinetic-Pharmacodynamic (PK-PD) Target Attainment Analyses to Support Ceftobiprole Continuous Infusion Dosing Regimens for Patients with Staphylococcus aureus Bacteremia (SAB)

**DOI:** 10.1093/ofid/ofaf695.179

**Published:** 2026-01-11

**Authors:** Sujata M Bhavnani, Jeffrey P Hammel, Christopher M Rubino, Karine Litherland, Kristie Zappas, Mark E Jones, Tony N Hodges, Marc Engelhardt, Rolf J Wagenaar

**Affiliations:** Institute for Clinical Pharmacodynamics, Schenectady, New York; Institute for Clinical Pharmacodynamics, Schenectady, New York; Institute for Clinical Pharmacodynamics, Schenectady, New York; Basilea Pharmaceutica International Ltd, Allschwil,, Basel-Landschaft, Switzerland; Innoviva Specialty Therapeutics, Inc., Waltham, Massachusetts; Basilea Pharmaceutica International Ltd., Allschwil, Switzerland, Allschwil,, Basel-Landschaft, Switzerland; Innoviva Specialty Therapeutics, Waltham, Massachusetts; Basilea Pharmaceutica International Ltd., Allschwil, Switzerland, Allschwil,, Basel-Landschaft, Switzerland; Basilea Pharmaceutica International Ltd, Allschwil,, Basel-Landschaft, Switzerland

## Abstract

**Background:**

Ceftobiprole medocaril, an intravenously administered cephalosporin that is rapidly converted to the active moiety ceftobiprole, received FDA approval in April 2024 for the treatment of adult patients with SAB, including infective endocarditis. Approved ceftobiprole dosing regimens adjusted based on creatinine clearance (CLcr), and administered as a 2-hour infusion, are PK-PD optimized [IDWeek 2023, Poster 2531]. PK-PD target attainment was evaluated for total 24-hour ceftobiprole dosing regimens administered as continuous infusion (CI) and compared to that for approved dosing regimens.

**Figure:**
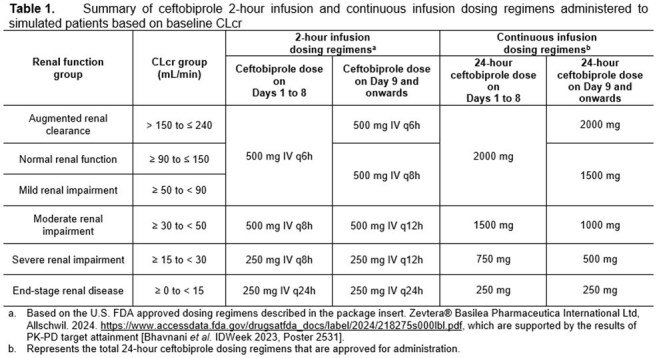

**Figure:**
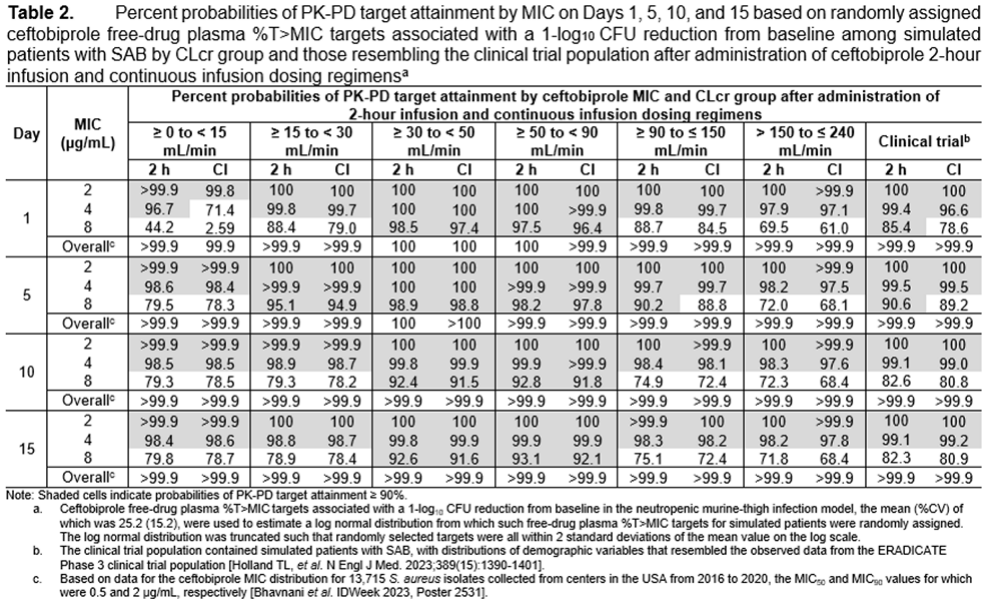

**Methods:**

Using a previously developed population PK model [IDWeek 2023, Poster 2561], randomly assigned free-drug plasma %T >MIC targets associated with a 1-log_10_ CFU reduction from baseline in a neutropenic murine-thigh infection model and *in vitro* surveillance data for *S. aureus*, and simulation, percent probabilities of PK-PD target attainment were evaluated for ceftobiprole CI dosing regimens. These dosing regimens were administered to over 3,000 simulated patients with SAB replicated from the ERADICATE Phase 3 clinical trial population [Holland *et al.,* NEJM 2023], evaluated with CLcr as observed or randomly assigned uniformly within specified ranges. Results were compared for ceftobiprole CI and approved dosing regimens summarized in Table 1.

**Figure:**
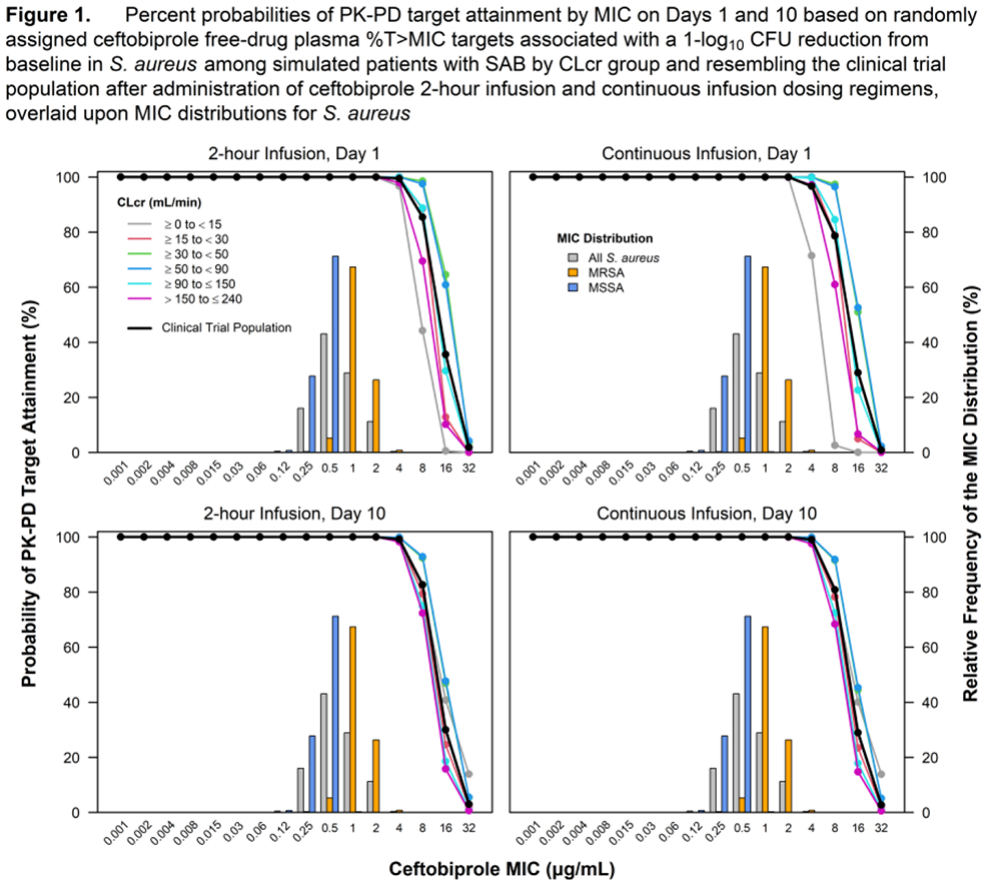

**Results:**

Percent probabilities of PK-PD target attainment by MIC on Days 1, 5, 10, and 15 shown in Table 2 and graphic results for Days 1 and 10, overlaid on *S. aureus* MIC distributions, shown in Figure 1 among simulated patients after administration of ceftobiprole CI and 2-hour infusion dosing regimens demonstrated comparable results at MIC values of interest. At an MIC of 2 µg/mL, the ceftobiprole susceptible breakpoint, percent probabilities of PK-PD target attainment on all days of assessment ranged from 99.8 to 100% for both sets of dosing regimens. At an MIC of 4 µg/mL, the intermediate breakpoint, percent probabilities were ≥ 96.6% for all assessments, except for simulated patients with CLcr ≥ 0 to < 15 mL/min after CI on Day 1.

**Conclusion:**

These PK-PD target attainment results provide support for the administration of ceftobiprole as CI for SAB. Clinical data describing patient outcomes will be useful to further support the use of ceftobiprole CI.

**Disclosures:**

All Authors: No reported disclosures

